# Transcriptomic and functional profiling of endothelial dysfunction induced by polystyrene nanoplastics

**DOI:** 10.3389/ftox.2026.1812922

**Published:** 2026-04-17

**Authors:** Joan Martín-Pérez, Aliro Villacorta, Javier Gutiérrez-García, Raquel Egea, Michelle Morataya-Reyes, Mireia Cassú-Casadevall, Irene Barguilla, Ricard Marcos, Alba Hernández, Alba García-Rodríguez

**Affiliations:** 1 Group of Mutagenesis, Department of Genetics and Microbiology, Faculty of Biosciences, Universitat Autònoma de Barcelona, Barcelona, Spain; 2 Facultad de Recursos Naturales Renovables, Universidad Arturo Prat, Iquique, Chile

**Keywords:** functional effects, genotoxicity, HUVEC, internalization, polystyrene nanoplastics, transcriptomics

## Abstract

**Background:**

The presence of micro- and nanoplastics (MNPLs) in human blood raises concerns about their vascular impact and their potential contribution to cardiovascular diseases. Endothelial cells are a primary target of circulating MNPLs; however, the molecular and functional consequences of this exposure remain largely undefined.

**Methods:**

In this study, we exposed primary human umbilical vein endothelial cells (HUVECs) to carboxylated polystyrene nanoplastics (PS-NPLs; 30, 50, and 100 nm) and integrated RNA sequencing with targeted functional assays.

**Results:**

Transcriptomics revealed a robust response characterized by coordinated dysregulation of cholesterol homeostasis, genotoxic stress and DNA repair, inflammatory signaling, and endothelial plasticity (endothelial-to-mesenchymal transition). Guided by these signatures, functional assays confirmed increased intracellular cholesterol, DNA damage, remodelling of migratory capacity and angiogenic behaviour, and reduced IL-6 secretion.

**Discussion:**

Overall, the concordance between transcriptomic programs and functional endpoints supports a mechanistic framework in which PS-NPL exposure rewires endothelial metabolic and stress-response networks, with downstream consequences for key vascular functions. Differences across the nanoscale range modulated the magnitude and temporal profile of specific endpoints, but the shared molecular core response predominated across treatments.

## 1 Introduction

Plastics are synthetic polymers predominantly composed of carbon–carbon (C–C) backbones. While a subset of biodegradable plastics is derived from natural feedstocks such as cellulose or cornstarch, the vast majority is derived from fossil resources (Rodrigues et al., 2022). Global plastic production has increased exponentially, from 2 million tons in 1950 to 359 million tons in 2018 (Shen et al., 2020). This expansion is driven by plastics’ unique combination of durability, chemical resistance, low water permeability, light weight, and cost-effectiveness, which has made them indispensable across packaging, consumer goods, medical devices, and construction (Oliveira et al., 2020; Domenech and Marcos, 2021). However, large-scale production, widespread use, and inadequate recycling have led to environmental accumulation (Hong, 2023). Their persistence to degradation allows plastics to accumulate in air, water, soil, and biota (Shen et al., 2020). Environmental processes such as fragmentation and photo-oxidation break plastics into smaller particles, generating secondary microplastics (<1 mm) and nanoplastics (<1 µm) (MNPLs). Moreover, MNPLs are deliberately produced for applications in cosmetics, drug delivery, and 3D printing inks (Facciolà et al., 2021; Rodrigues et al., 2022).

Beyond environmental pollution, MNPLs pose emerging human health risks owing to their ability to enter the body primarily via ingestion and inhalation (Rubio et al., 2020; O’Neill and Lawler, 2021). Their nanoscale dimensions enable them to traverse biological barriers, such as the gastrointestinal and respiratory epithelia (Lehner et al., 2019; Domenech et al., 2020), reach the systemic circulation, and accumulate in diverse tissues (Rajendran and Chandrasekaran, 2023). Indeed, MNPLs have been detected in human tissues/organs, including the placenta, lungs, liver, breast milk, and blood (Ali et al., 2024). In human blood, plastic particles ≥700 nm at concentrations averaging 1.6 μg/mL were reported with polystyrene (PS) among the most abundant polymers (Leslie et al., 2022). In blood vessels, MNPLs may interact with vascular endothelial cells, potentially contributing to cardiovascular disease (CVD). In line with this, Marfella et al. (2024) demonstrated that the presence of MNPL in carotid plaques increased the risk of myocardial infarction and stroke, providing a possible mechanistic link between exposure and CVD. Understanding the toxicity of nanoplastics (NPLs) in endothelial cells is therefore crucial for evaluating their cardiovascular impact. Among available models, human umbilical vein endothelial cells (HUVECs) are widely used owing to their primary human origin and their ability to recapitulate key features of the vascular endothelium (Cao et al., 2017). Prior studies have demonstrated that HUVECs efficiently internalize NPLs, eliciting responses such as cytotoxicity, oxidative stress, and autophagy (Cao et al., 2017; Martín-Pérez et al., 2024). However, most evidence remains fragmented across individual endpoints, and omics-level signatures are rarely connected to specialized endothelial functions. To address this gap, we combined transcriptomic profiling (RNA-seq; DEG, ORA, and GSEA) with functional assays that capture core features of endothelial dysfunction, including cholesterol accumulation (Filipin III staining), genome integrity (comet assay), migration (wound-healing assay), angiogenic behaviour (tube formation assay), and inflammatory signaling (IL-6 ELISA). We included three PS-NPL sizes (30, 50, and 100 nm) to assess whether the molecular programs and functional consequences identified are robust across the nanoscale range. This integrated approach provides mechanistic insight into how PS-NPLs modulate primary human endothelial cells and strengthens the biological interpretation of transcriptomic findings beyond their use as a screening layer for hazard identification.

## 2 Materials and methods

### 2.1 Sources of NPLs

Fluorescently labeled (FL) PS-C-NPLs of 30 nm (L5155; nominal size: 0.02–0.04 μm) were obtained from Sigma-Aldrich (Steinheim, Germany). For the 50 nm NPLs, both fluorescently labeled (16661-10; mean diameter: 0.05 μm) and non-labeled (NL) (15913-10; mean diameter: 0.05 μm) PS-C-NPLs were purchased from Polysciences Inc. (Warrington, PA, USA). Similarly, for the 100 nm NPLs, fluorescently labeled (16662-10; mean diameter: 0.10 μm) and non-labeled (16688-15; mean diameter: 0.10 μm) PS-C-NPLs were obtained from the same supplier.

### 2.2 PS-C-NPLs characterization

Characterization of the PS-C-NPLs was performed by diluting the dispersions in Milli-Q water and/or Endothelial Cell Growth Medium 2 (EGM-2; Promocell; Heidelberg, Germany). For dry-state transmission electron microscopy (TEM) analysis, PS-C-NPLs were prepared at a concentration of 200 μg/mL in Milli-Q water. Copper grids with a carbon coating were immersed in the working solutions, dried overnight, and imaged using a JEOL JEM 1400 TEM (JEOL Ltd., Tokyo, Japan) at 120 kV. Martin diameters of 100 individual NPLs were measured in ImageJ v1.8.0_172 to determine size distributions. For colloidal behaviour, PS-C-NPLs (100 μg/mL) were suspended in both Milli-Q water and EGM-2. Particle size and Z-potential were measured using a Zetasizer® Ultra (Malvern Panalytical, Cambridge, UK) with appropriate cuvettes. Each sample was analysed in triplicate.

### 2.3 Cell line and culture conditions

Human umbilical vein endothelial cells (HUVECs, single donor; C-12200) were obtained from Promocell (Heidelberg, Germany). Derived from the endothelial lining of umbilical cord veins, HUVECs are widely recognized as a representative primary model of the human endothelium for physiological and toxicological studies. Cells were cultured in EGM-2 medium (C-22011, Promocell) in collagen-coated flasks prepared with rat tail collagen I (Corning Inc., New York, NY, USA) at 5 μg/cm^2^. Cultures were maintained at 37 °C in a humidified 5% CO_2_ atmosphere with medium changes every other day, and experiments were performed with cells up to passage 6.

### 2.4 PS-C-NPL treatments

Cells were exposed to PS-C-NPLs diluted in EGM-2 at 100 μg/mL for 24 h, unless specified otherwise. To ensure comparable cell density, treatments with different durations were applied in a staggered manner and analyzed simultaneously. Cells were typically seeded at 26,300 cells/cm^2^ in collagen I-coated wells (5 μg/cm^2^) and cultured for 48 h before the medium was replaced with the corresponding treatment. Untreated cells served as negative controls.

### 2.5 Cytotoxicity of PS-C-NPLs

Cytotoxicity of PS-C-NPLs in HUVECs was assessed using the Coulter principle with a Beckman Coulter Z1-D cell counter (Beckman Coulter Inc., Pasadena, CA, USA). Cells were exposed to PS-C-NPLs (30, 50, and 100 nm) at 50, 100, and 200 μg/mL for 24 h. Viability was calculated as the mean cell count in each treatment relative to the untreated control, expressed as a percentage. Experiments were performed in triplicate with technical duplicates analyzed for each condition.

### 2.6 RNA isolation and sequencing

After 24 h exposure to 100 μg/mL PS-C-NPLs (30, 50, and 100 nm), RNA was isolated using the TRI Reagent® protocol (Merck, Darmstadt, Germany). Lysates were processed with chloroform extraction and 2-propanol precipitation, and RNA pellets were washed with 75% ethanol before resuspension in RNase-free water. RNA concentration and purity were determined with a NanoDrop® spectrophotometer (Thermo Fisher Scientific, Waltham, MA, USA). Samples were submitted to Macrogen (Seoul, South Korea) for RNA sequencing (RNA-seq). Experiments were performed with technical quadruplicates for each treatment and triplicates for the negative control.

### 2.7 Bioinformatic analysis for transcriptomic data

Statistical analyses were performed in R version 4.3.2 (R Core Team, 2023) using RStudio (Posit Team, 2023). Raw FASTQ files were quality filtered with Rfastp (Wang and Carroll, 2024) to remove adapters, trim low-quality bases, and discard reads shorter than 20 bp. Filtered reads were mapped to the human genome GRCh38 (GENCODE) and counted with Rsubread (Liao et al., 2019; Carlson, 2023). Lowly expressed genes were removed with the filterByExpr function from edgeR, and normalization factors were calculated with calcNormFactors (Law et al., 2016; Chen et al., 2024). Surrogate variable analysis (sva; Leek et al., 2023) was applied to account for technical variation, and differential expression was performed with limma-voom (Law et al., 2014; Ritchie et al., 2015). Differentially expressed genes (DEGs) were obtained by contrasting treated samples with the negative control. Functional enrichment was assessed using clusterProfiler (Wu et al., 2021; Dolgalev, 2022) by both over-representation analysis (ORA) and gene set enrichment analysis (GSEA), with GO Cellular Component and MSigDB hallmark (H) collections as references. Parameters were left as default, except for the maximum gene set size in GO GSEA, which was set to 800. ORA further analyzed unique and shared DEGs across treatments with the MSigDB hallmark collection. Data visualization was performed with ggplot2, eulerr, and ComplexHeatmap (Wickham, 2016; Gu, 2022; Larsson, 2024).

### 2.8 Cellular uptake by flow cytometry

The internalization of PS-C-NPLs by HUVECs was evaluated by flow cytometry. Cells were treated with 100 μg/mL of FL PS-C-NPLs (30, 50, and 100 nm) for 20 min, 2 h, 12 h, and 24 h. Viable cells were stained with Via-Probe™ Red Nucleic Acid Stain (1:200; BD Biosciences, Franklin Lake, NJ, USA). Fluorescence was measured using a CytoFLEX flow cytometer (Beckman Coulter, Pasadena, CA, USA) with excitation/emission wavelengths of 488/525 nm for FL PS-C-NPLs and 638/660 nm for Via-Probe™. Two parameters were quantified: (i) the percentage of cells internalizing PS-C-NPLs and (ii) the relative amount internalized per cell. For each condition, 10,000 cells were analyzed using Cytexpert software (Beckman Coulter, Pasadena, CA, USA). Experiments were performed in triplicate, and technical duplicates were analyzed for each replicate.

### 2.9 Cellular uptake assessment by confocal microscopy

Laser confocal microscopy was used to confirm the localization of FL PS-C-NPLs (30, 50, and 100 nm) within HUVECs. After exposure, the media were removed, and cells were incubated with Hoechst 33342 (1:500) and CellMask™ Deep Red (1:500) (ThermoFisher Scientific, Waltham, MA, USA) to stain nuclei and membranes, respectively. Images were acquired with a Leica TCS SP5 confocal microscope (Leica Microsystems GmbH, Mannheim, Germany). FL PS-C-NPLs were detected inside the cells at 488 nm, nuclei at 405 nm, and membranes at 633 nm. Several random fields were imaged per sample, and images were processed using ImageJ v1.8.0_172.

### 2.10 Cellular uptake assessment by TEM

TEM was used to complement flow cytometry and confocal microscopy by providing high-resolution localization of PS-C-NPLs and identifying associated subcellular structures. After 24 h of exposure to PS-C-NPLs (30, 50, and 100 nm), cells were collected, centrifuged, and fixed in 2.5% (v/v) glutaraldehyde (Merck, Darmstadt, Germany) and 2% (w/v) paraformaldehyde (EMS, Hatfield, PA, USA) in 0.1 M cacodylate buffer (pH 7.4; Sigma-Aldrich, Steinheim, Germany). Samples were processed following standard TEM protocols (Annangi et al., 2015). Cells were post-fixed with osmium tetroxide, dehydrated through a graded acetone series, and embedded in Eponate 12™ resin (Ted Pella Inc., Redding, CA, USA), polymerized at 60 °C. Ultrathin sections were stained with uranyl acetate and Reynolds lead citrate and examined with a JEOL JEM 1400 TEM (JEOL Ltd., Tokyo, Japan) equipped with an ES1000W Erlangshen CCD camera (GATAN Inc., Pleasanton, CA, USA).

### 2.11 Genotoxicity by the comet assay

The genotoxic potential of PS-C-NPLs (30, 50, and 100 nm) in HUVECs was evaluated by the comet assay to detect DNA strand breaks (Collins et al., 2023). Cells were exposed to PS-C-NPLs for 2 and 24 h, with methyl methanesulfonate (MMS; 200 μM, 30 min at 37 °C; Sigma-Aldrich, St. Louis, MO, USA) as the positive control. After exposure, cells were centrifuged (150 g, 6 min, 4 °C), resuspended in PBS (1 × 10^6^ cells/mL), and mixed with 0.75% low-melting agarose (1:10). Drops of 7 µL were placed on Gelbond® film (Lonza Bioscience, Basel, Switzerland) in triplicate. Following overnight lysis at 4 °C, DNA was unwound in electrophoresis buffer (35 min, 4 °C) and electrophoresed (20 V, 300 mA, 20 min, 4 °C). Films were washed, fixed in ethanol for 1 h, and air-dried overnight. Samples were stained with SYBR™ Gold (20 min, 1:10,000; Invitrogen, Waltham, MA, USA) and visualized using an Olympus BX50 epifluorescence microscope (Olympus, Tokyo, Japan) at ×20 magnification. DNA damage was quantified as % tail DNA using Komet 5.5 software (Kinetic Imaging Ltd., Liverpool, UK), with 100 cells per sample analyzed. Each experimental condition was assessed using two samples per replicate, and the entire experiment was performed in duplicate.

### 2.12 Cholesterol detection via Filipin III staining

HUVECs were seeded in µ-Plate 24 Well black plates (ibidi, Gräfelfing, Germany), and intracellular cholesterol was assessed by Filipin III staining. After 24 h exposure to PS-C-NPLs (30, 50, and 100 nm), cells were fixed with an optimized two-step protocol to preserve morphology. Cells were pre-fixed with a 1:1 mixture of EGM-2 and 4% paraformaldehyde (PFA) at 37 °C for 10 min, followed by 4% PFA at room temperature for 10 min. After PBS washes, aldehyde groups were quenched with 1.5 mg/mL glycine in PBS. Filipin III (SAE0087, Sigma-Aldrich, Steinheim, Germany) was diluted in PBS containing 10% FBS to 0.05 mg/mL and applied for 2 h at room temperature in the dark. Images were acquired using a Zeiss LSM 980 confocal microscope (Carl Zeiss Microscopy GmbH, Jena, Germany) at ×20 magnification (excitation 353 nm, emission 465 nm), with five images per well. Fluorescence intensity was quantified in 400 cells (20 per image) using ImageJ (v1.8.0_172) with manual ROIs, across two biological replicates with two technical replicates each. Raw Integrated Density values were normalized to cell area.

### 2.13 Wound healing assay

To evaluate the effects of PS-C-NPLs (30, 50, and 100 nm) on HUVEC migration, a wound-healing assay was performed using Culture-Inserts 2 Well (Ibidi GmbH, Gräfelfing, Germany) according to the manufacturer’s protocol. Inserts were placed in collagen-coated wells, and 21,000 cells were seeded in each compartment with EGM-2 medium containing 100 μg/mL PS-C-NPLs. After 24 h of incubation, the inserts were removed, the wells were washed, and fresh medium at the same NPL concentration was added. Images were captured at 0 h with a Zeiss Axio Observer A1 inverted microscope (Carl Zeiss Microscopy GmbH, Jena, Germany) using a ×10 objective, and additional image were acquired every 2h for up to 8 h. Wound closure was quantified with ImageJ (v.8.0_172) using the “Wound Healing Size Tool” plug-in (Surez-Arnedo et al., 2020), applying the formula $Wound\;closure\;\%=\left(\frac{A_{t=0}-A_{t=\Delta t}}{A_{t=0}}\right)x100\%$ , where A_t=0_ is the initial wound area and A_t=Δt_ the area at each time point. Experiments were repeated three times with technical triplicates per condition.

### 2.14 Tube formation assay

An angiogenesis assay was performed to assess the effects of PS-C-NPLs (30, 50, and 100 nm) on HUVEC tube formation. After 48 h incubation, passage 5 cells were treated for 24 h with 100 μg/mL PS-C-NPLs. Then, 10,000 cells were seeded into µ-Slide 15 Well 3D plates (Ibidi GmbH, Gräfelfing, Germany) pre-coated with Corning® Matrigel® Matrix (Corning, NY, USA) in medium containing the same concentration of PS-C-NPLs. Positive controls were treated with PTPase inhibitor/Suramin (Abcam, Cambridge, UK) at 10–20 µM. After 14 h of incubation, cells were washed, and tube formation was evaluated using a Zeiss LSM 980 confocal microscope (Carl Zeiss, Jena, Germany) in brightfield mode. For each well, 12 stitched Z-stack images were acquired at ×10 magnification with extended depth of focus. Quantitative parameters, including covered area, total tube length, and branching points, were analyzed with the WimTube image analysis service (Wimasis Image Analysis, Onimagin Technologies SCX, Córdoba, Spain). The experiment was repeated three times with technical quadruplicates per condition.

### 2.15 IL-6 ELISA (enzyme-linked immunosorbent assay)

IL-6 levels were quantified by ELISA in culture supernatants collected after 24 h of treatment with PS-C-NPLs (30, 50, and 100 nm). Supernatants were processed using ELISA kits (KHC0061, Invitrogen; Waltham, MA, USA) according to the manufacturer’s instructions. Absorbance was measured at 450 nm, and standard curves were generated with a 4-parameter logistic fit. IL-6 concentrations were calculated for each treatment. Experiments were performed three times with technical triplicates per condition.

### 2.16 Statistical analysis for *in vitro* assays

Data analysis was conducted with GraphPad Prism 9 (GraphPad Software Inc., CA, USA). Normality was assessed with the Shapiro–Wilk test. For normally distributed data, one-way ANOVA was performed, followed by Dunnett’s test to compare each treatment with the control (CTL vs. PS-C 30 nm, CTL vs. PS-C 50 nm, CTL vs. PS-C 100 nm) and Tukey’s test to compare treatments with each other (PS-C 30 nm vs. PS-C 50 nm, PS-C 30 nm vs. PS-C 100 nm, PS-C 50 nm vs. PS-C 100 nm). For non-parametric data, the Kruskal–Wallis test followed by Dunn’s test was used. Statistical significance was defined as *p ≤ 0.05, **p ≤ 0.01, and ***p ≤ 0.001. Data is presented in figures as mean ± standard error of the mean (SEM).

## 3 Results and discussion

### 3.1 Physicochemical characterization of PS-C-NPLs

**FIGURE 1:**
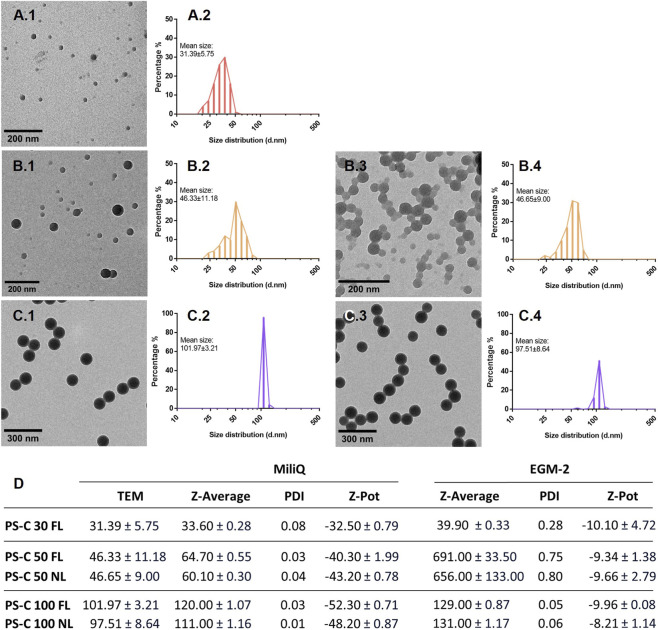
Physicochemical characterization of PS-C-NPLs. Panels **(A)** correspond to PS-C-NPLs of 30 nm, panels **(B)** to PS-C-NPLs of 50 nm, and panels **(C)** to PS-C-NPLs of 100 nm. Panels **(A.1–C.1)** show TEM images of fluorescently labeled NPLs, with panels **(A.2–C.2)** displaying the corresponding size distribution histograms. Panels **(B.3,C.3)** present TEM images of non-labeled NPLs, with panels **(B.4,C.4)** showing the respective histograms. Panel **(D)** summarizes TEM-derived Martin’s diameter (nm), hydrodynamic diameter (d.nm), and ζ-potential (mV) values obtained by TEM and DLS in Milli-Q water and EGM-2.

PS-C-NPLs of 30, 50, and 100 nm were selected to span a representative nanoscale range and to test the robustness of endothelial responses across particle sizes. TEM imaging confirmed that all three sizes maintained a spherical morphology in Milli-Q water, with diameters consistent with manufacturer specifications (Figures 1A–C). Hydrodynamic size measurements in Milli-Q water (Figure 1D) were close to nominal values for all NPLs. In EGM-2, PS-C 30 and 100 nm maintained Z-average sizes comparable to those in Milli-Q water. In contrast, PS-C 50 nm showed a pronounced size increase (650–690 nm) with high PDI (polydispersity indices) (0.75–0.80), indicating aggregation of both FL and NL counterparts. Importantly, DLS number-based distributions rather than intensity-based demonstrated that the predominant fraction of PS-C 50 nm in EGM-2 remained near 50 nm (Supplementary Figures S1B.1,B.2), and submicrometric fraction analysis confirmed that >99% of particles were below 200 nm in both cases. Thus, the elevated Z-average reflected a minor fraction of aggregates dominating the scattering signal. Surface charge analysis (Figure 1D) showed modest differences in Milli-Q water (−32.5 to −52.3 mV), but in EGM-2 all PS-C-NPLs displayed comparable ζ-potentials (−8.2 to −10.1 mV), consistent with previous observations (Santos et al., 2011; Paget et al., 2015; Banerjee et al., 2021). These values reflect partial charge screening by serum proteins forming a protein corona, thereby reducing surface charge differences among particles (da Silva et al., 2019). Overall, the characterization supports that the three PS-C-NPL preparations behave as expected under exposure conditions and provides a consistent basis for comparing molecular and functional responses across the nanoscale range under similar surface electrostatics.

### 3.2 Viability assessment of HUVECs exposed to PS-C-NPLs

Assessment of HUVEC viability after 24 h exposure to PS-C-NPLs (50–200 μg/mL) showed no significant cytotoxicity across particle sizes, with only a slight, non-significant reduction at 200 μg/mL (Supplementary Figure S2). These findings, consistent with previous reports in HUVECs (Martín-Pérez et al., 2024), indicate good acute tolerability and confirm that downstream alterations are not attributable to loss of viability. Accordingly, 100 μg/mL was selected as a sublethal concentration for subsequent assays, ensuring adequate exposure while preserving cellular integrity. This concentration is consistent with standard practice in *in vitro* nanotoxicology for hazard identification (Martín-Pérez et al., 2024; Lu et al., 2022; Lee et al., 2021; Brouwer et al., 2025). Furthermore, it is particularly justified given that current analytical methods cannot yet reliably quantify the nanometric fraction in biological matrices, leaving baseline circulating levels largely unknown. Moreover, local nanoplastic concentrations at the endothelial surface may be substantially higher than average blood levels due to tissue-level bioaccumulation, especially within areas of plaque formation (Marfella et al., 2024).

### 3.3 Transcriptomic profiling of HUVECs under PS-C-NPL exposure

**FIGURE 2:**
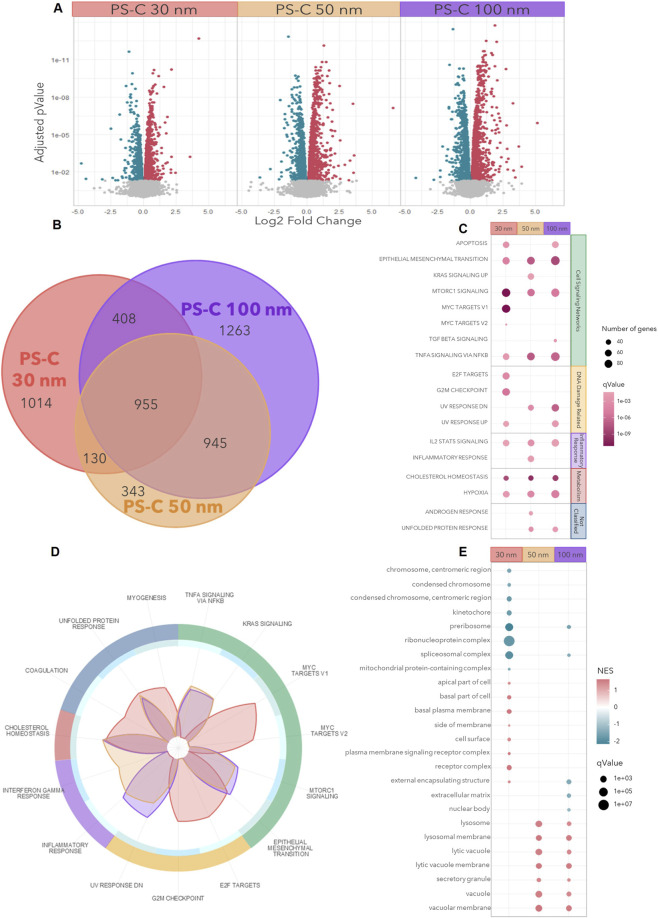
RNA-seq bioinformatic analyses of HUVECs exposed to PS-C-NPLs. **(A)** Volcano plots show gene-expression changes for each treatment, with upregulated genes shown in red and downregulated genes in blue. **(B)** Venn diagram illustrates the overlap and unique sets of DEGs across the different particle sizes. **(C)** Over-representation analysis (ORA, hallmark collection) identifies enriched pathways grouped into signaling, DNA damage, inflammation, metabolism, and unclassified categories. **(D)** Gene set enrichment analysis (GSEA, hallmark collection) is represented as radar plots of absolute NES values, highlighting enrichment patterns across treatments. **(E)** GSEA of Gene Ontology cellular components. PS-C 30 nm in red, PS-C 50 nm in orange, and PS-C 100 nm in purple.

RNA-seq analysis revealed that all PS-C-NPL treatments induced extensive transcriptional alterations in HUVECs (Figure 2A). PS-C 100 nm triggered the largest number of DEGs (3,571), followed by PS-C 30 nm (2,507) and PS-C 50 nm (2,373). Despite these differences in magnitude, 955 DEGs were shared across all treatments, delineating a core transcriptomic response to PS-C-NPL exposure (Figure 2B). Overlap analysis indicated condition-specific components, with more unique DEGs in PS-C 30 nm and greater overlap between PS-C 50 and 100 nm.

Functional enrichment analyses highlighted a dominant shared response and additional treatment-associated signatures. ORA of hallmark gene sets (Figure 2C; MSigDB) revealed a consistent core across all treatments, with “Cholesterol homeostasis” and “Epithelial–mesenchymal transition” as the most significantly enriched pathways, together with “IL-2 STAT5 signaling,” “mTORC1 signaling,” “TNFα signaling via NF-κB,” and “Hypoxia.” These findings define a shared axis of metabolic, inflammatory, and signaling-related alterations across the nanoscale range. Beyond this core, some enrichment patterns differed between conditions: PS-C 30 nm showed stronger enrichment of genome integrity–related programs coupled to cell-cycle control (e.g., “MYC targets,” “E2F targets,” “G2M checkpoint”), PS-C 50 nm was more closely associated with inflammatory signatures (“Inflammatory response”), and PS-C 50/100 nm showed enrichment of genome integrity–linked stress programs consistent with DNA damage and repair engagement (e.g., “UV response DN”). In addition, “UV response UP” was enriched in PS-C 30 and 100 nm, further supporting shared DNA damage–response/repair-associated transcriptional engagement across these conditions. GSEA corroborated these results, consistently recovering the core responses “Cholesterol homeostasis” and “Epithelial–mesenchymal transition,” while also capturing modest condition-associated shifts in enrichment patterns (Figure 2D).

Finally, GSEA on cellular components (GO) revealed treatment-associated differences in the subcellular structures implicated (Figure 2E). PS-C 30 nm showed relatively stronger enrichment for DNA and cell division complexes (“chromosome, centromeric region,” “condensed chromosome,” “kinetochore”), RNA transcription and protein synthesis machinery (“preribosome,” “ribonucleoprotein complex,” “spliceosomal complex”), and membrane-related structures (“apical part of cell,” “basal part of cell,” “basal plasma membrane,” “cell surface,” “external encapsulating structure”). In contrast, PS-C 50 and 100 nm were more prominently associated with vacuolar and lysosomal structures (“lysosome,” “lytic vacuole,” “secretory granule,” “vacuolar membrane”). Such patterns may be consistent with differences in intracellular distribution and processing pathways, thereby providing a rationale for subsequent flow cytometry, confocal microscopy, and TEM analyses of particle uptake and ultrastructural localization.

Together, these transcriptomic results provide a pathway-level map of endothelial responses to PS-C-NPL exposure. A robust core program emerged across treatments, centered on cholesterol metabolism, endothelial plasticity (EMT), inflammatory signaling, and stress-response networks, with genome integrity control emerging as a prominent additional axis across the dataset. Importantly, the convergence between ORA and GSEA allowed us to use the bioinformatic readouts as a hypothesis-generating and mechanistic layer to guide targeted phenotyping. We therefore examined NPL internalization and intracellular distribution (flow cytometry, confocal microscopy, and TEM), then assessed genome integrity (comet assay), cholesterol accumulation (Filipin III staining), endothelial migration (wound-healing assay), angiogenic behaviour (tube formation assay), and inflammatory signaling (IL-6 secretion).

### 3.4 Internalization and intracellular distribution of PS-C-NPLs in HUVECs

**FIGURE 3:**
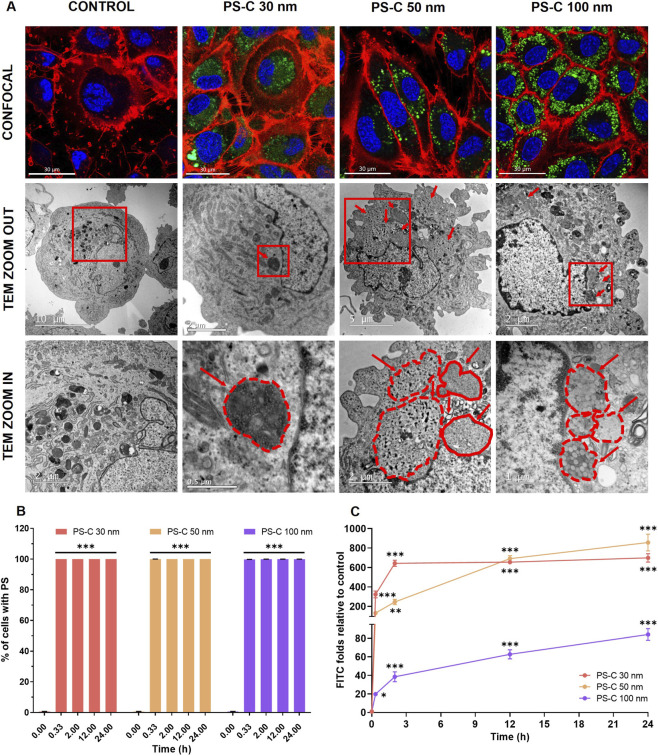
Internalization of PS-C-NPLs in HUVECs exposed to 100 μg/mL for 24 h. **(A)** Confocal microscopy shows labelled PS-C-NPLs (green), nuclei stained with Hoechst 33342 (blue), and cell membranes with CellMask™ (red). TEM images display vesicular structures containing PS-C-NPLs (red arrows), with magnified regions indicated by solid red squares and vesicle boundaries outlined by red dashed or continuous lines. **(B)** Flow cytometry quantification of the percentage of HUVECs internalizing PS-C-NPLs of 30, 50, and 100 nm. **(C)** Uptake kinetics represented as FITC fluorescence fold change vs. untreated controls at 20 min, 2 h, 12 h, and 24 h. Data are expressed as mean ± SEM. Statistical analysis was performed with one-way ANOVA with Dunnett’s post-test (parametric data). *p ≤ 0.05, **p ≤ 0.01, ***p ≤ 0.001.

Flow cytometry showed that HUVECs rapidly internalized fluorescent PS-C-NPLs across all conditions, with near-complete uptake within the first 20 min (Figure 3B). This uniform internalization at the population level provides a robust basis to connect molecular perturbations with downstream functional phenotypes. While overall uptake efficiency was comparable, kinetic and imaging readouts indicated differences in intracellular handling across the nanoscale range (Figures 3A–C). Confocal microscopy revealed widespread cytoplasmic accumulation, and TEM provided ultrastructural evidence of vesicular sequestration. PS-C 30 nm showed fewer vesicles and a more diffuse fluorescence pattern, suggestive of a larger fraction of dispersed particles. In contrast, PS-C 50 and 100 nm were predominantly associated with abundant vesicular compartments, consistent with an endolysosomal processing route. Supporting this interpretation, endolysosomal involvement in the processing of PS-NPLs in this size range (≥50 nm) has been previously reported: in rat basophilic leukaemia (RBL-2H3) cells, PS-NPLs of 50 and 500 nm accumulated within lysosomes (Liu et al., 2021). Importantly, these cellular phenotypes aligned with the bioinformatic signatures. GO cellular component GSEA highlighted plasma membrane/cell-surface–associated structures in PS-C 30 nm and vacuolar/lysosomal compartments in PS-C 50 and 100 nm. Overall, this subcellular enrichment pattern, together with imaging readouts, suggests differential internalization routes and subsequent intracellular handling across conditions. These trafficking features provide a mechanistic basis for interpreting RNA-seq programs and contextualizing downstream functional outcomes, including cholesterol dysregulation.

### 3.5 Genotoxicity of PS-C-NPLs by the comet assay

**FIGURE 4:**
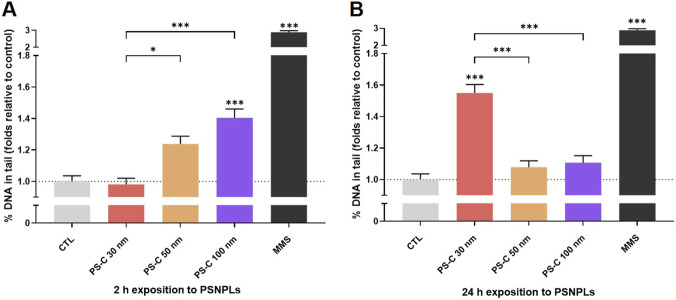
Genotoxic damage in HUVECs was assessed using the comet assay following 2-h **(A)** and 24-h **(B)** exposures to PS-C-NPLs at 100 μg/mL. MMS at 200 μM served as the positive control. Data are presented as mean ± SEM. Statistical analyses were performed using Kruskal–Wallis with Dunn’s post-test (non-parametric data). Statistical significance is denoted as *p ≤ 0.05, **p ≤ 0.01, and ***p ≤ 0.001.

The comet assay revealed genotoxic effects of PS-C-NPLs in HUVECs across multiple time points (Figure 4). After 2 h (Figure 4A), PS-C 100 nm significantly increased DNA strand breaks (40% above control), and PS-C 50 nm showed a similar trend (24% above control). At 24 h (Figure 4B), DNA damage was significantly increased only in the PS-C 30 nm condition (55% above control), indicating distinct temporal profiles across treatments. These data, together with the transcriptomic signatures previously described, suggest that PS-C-NPL exposure can elicit early DNA lesions and/or delayed genotoxic stress, with damage persistence reflecting the balance between lesion formation and activation of repair and cell-cycle control pathways.

The temporal profiles observed here are consistent with differential engagement of DNA repair and cell-cycle control responses. The early, transient increases observed for PS-C at 50/100 nm are consistent with activation of repair systems that resolve initial lesions (Roursgaard et al., 2022). In line with this interpretation, ORA and GSEA identified deregulation of UV response/DNA repair-related pathways for PS-C 50 and 100 nm (“UV response DN”), and ORA also detected “UV response UP” enrichment for PS-C 100 nm, suggesting a stronger induction of repair-associated transcriptional programs under this condition. In contrast, PS-C 30 nm showed less prominent enrichment of these repair-related signatures, while preferentially enriching cell-cycle and chromosome-associated pathways (E2F targets, G2M checkpoint; e.g., condensed chromosome, kinetochore). Together, these data support a mechanistic model in which PS-NPL exposure perturbs genome integrity and, depending on intracellular processing, can shift the response toward either repair-driven resolution or sustained stress coupled to cell-cycle dysregulation.

### 3.6 Intracellular cholesterol levels in HUVECs exposed to PS-C-NPLs by Filipin III staining

**FIGURE 5:**
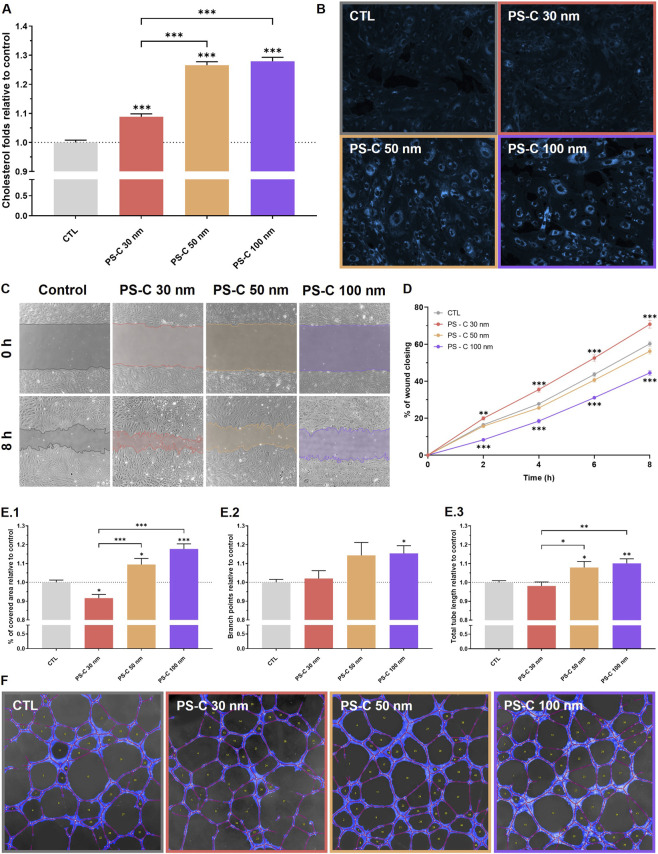
Effects of PS-C-NPLs on intracellular cholesterol, wound healing, and angiogenesis in HUVECs exposed to 100 μg/mL for 24 h. **(A,B)** Intracellular cholesterol: **(A)** Quantification of intracellular cholesterol levels after treatment with PS-C-NPLs. **(B)** Representative fluorescence microscopy images showing cholesterol staining with Filipin III in blue. **(C,D)** Wound healing assay: **(C)** Representative images showing wound closure at 0 and 8 h after treatment with PS-C-NPLs. **(D)** Quantification of wound closure dynamics (% of closure) over 8 h, measured at 2-h intervals. **(E,F)** Angiogenesis assay: **(E.1–3)** Quantitative analysis of angiogenesis potential, including **(E.1)** % of covered area relative to control, **(E.2)** number of branch points relative to control, and **(E.3)** total tube length relative to control. **(F)** Representative images of angiogenesis assays for each condition. Tubes are labelled in red, the covered area is highlighted in blue, and the number of loops is annotated within each loop, as analysed using Wimasis. Data are presented as mean ± SEM. Statistical analysis was performed using one-way ANOVA with Dunnett’s or Tukey’s post-tests for normally distributed data, and Kruskal–Wallis with Dunn’s post-test for non-parametric data. Statistical significance is indicated as *p ≤ 0.05, **p ≤ 0.01, and ***p ≤ 0.001.

Filipin III staining revealed increased intracellular cholesterol accumulation in HUVECs treated with PS-C-NPLs compared to controls (Figures 5A,B). Quantification of 400 individual cells confirmed significant increases across treatments. The magnitude of accumulation differed between conditions (9% for PS-C 30 nm and 27%–28% for PS-C 50/100 nm). However, the directionality was consistent, supporting cholesterol dysregulation as a central functional phenotype of PS-NPL exposure. This observation was directly supported by transcriptomic data, in which cholesterol homeostasis emerged as one of the most significantly enriched hallmarks across conditions.

Animal studies in mice have previously shown that PS-NPLs of different sizes (80 and 200 nm) can disrupt lipid metabolism, increasing hepatic triglyceride and cholesterol accumulation (Fan et al., 2022; Yu et al., 2024), but effects on human cellular models have not yet been examined. Our results extend this knowledge, demonstrating that PS-C-NPLs induce intracellular cholesterol accumulation in HUVECs. This core response was corroborated by transcriptomic analyses, where ORA and GSEA identified cholesterol homeostasis as one of the most significantly dysregulated pathways across treatments. Interestingly, more than half of the genes within this pathway (Supplementary Figure S3A) were significantly altered, displaying a consistent upregulation pattern. Among the altered genes, *SREBF2* and its downstream effectors (*MVD*, *LDLR*, *HMGCR*) were upregulated (data not shown), suggesting that activation of cholesterol biosynthesis contributes to the observed accumulation (Fisher et al., 2021).

Additionally, qualitative analysis of Filipin III images also revealed differences in cholesterol localization. In control cells, cholesterol displayed a perinuclear distribution, reflecting synthesis in the endoplasmic reticulum (Lyu et al., 2017). In contrast, PS-C-treated cells exhibited cytoplasmic vesicular accumulation, particularly evident in the 50 and 100 nm groups. Similar accumulation patterns have been reported in HUVECs when cholesterol trafficking is blocked by inhibitors such as cepharanthine, resulting in late endolysosomal retention (Lyu et al., 2017). Supporting this, our cellular component GSEA identified lysosomal alterations in PS-C at 50 and 100 nm, corresponding to the treatments with the largest increases in cholesterol. Together, these findings indicate that PS-C-NPL internalization may interfere with lysosomal function, disrupting cholesterol trafficking and promoting intracellular accumulation.

Intracellular cholesterol accumulation in the endothelium is associated with endothelial dysfunction and early atherogenic events (Hassan et al., 2006; Baumer et al., 2017; Higashi, 2023). Reported consequences include cholesterol crystal formation, TNF-α–mediated apoptosis, cellular senescence, enhanced ROS production, and impaired vasodilation (Hassan et al., 2006; Baumer et al., 2017; Fisher et al., 2021; Ye et al., 2023; Ziegler et al., 2024). Importantly, alterations in cholesterol trafficking also influence membrane fluidity, intracellular transport, signaling, migration, and angiogenic capacity (Lyu et al., 2017). Based on the pronounced cytoplasmic accumulation observed here, we next assessed endothelial migration and angiogenesis, two key specialized functions of endothelial cells (Lee et al., 2021).

### 3.7 Migration of HUVECs exposed to PS-C-NPLs by wound healing assay

The wound healing assay showed that PS-C-NPL exposure remodelled HUVEC migratory behaviour (Figures 5C,D). At 8 h, PS-C 30 nm modestly increased wound closure (70.8% vs. 60.2% in controls), whereas PS-C 100 nm impaired migration (44.5% closure). PS-C 50 nm produced an intermediate effect that did not reach significance. These results indicate that nanoscale PS-NPL exposure can shift endothelial motility in different directions depending on the overall cellular response context.

Most studies have reported that MNPL exposure impairs cell migration across diverse models, including HUVECs, bronchial and alveolar epithelial cells, and trophoblasts, with PS-MNPLs (30–1,000 nm) reducing migratory capacity (Lee et al., 2021; Hu et al., 2022; Wan et al., 2024; Lv et al., 2024). Moreover, abnormal cholesterol accumulation has been linked to reduced HUVEC migration (Lyu et al., 2017), consistent with our finding that PS-C at 100 nm induced the largest cholesterol increase and impaired migration. Nevertheless, enhanced migration has also been described in HUVECs following MNPL exposure. Traversa et al. (2024) reported that polyethylene MNPLs of 200 and 9,900 nm significantly increased migration, indicating that the stimulatory effect observed here for PS-C 30 nm is not unprecedented and may represent an alternative response of endothelial cells to NPL exposure.

Our transcriptomic data further corroborates these functional outcomes. Consistent with Traversa et al. (2024), who observed alterations of the EMT pathway in HUVECs after polyethylene exposure, our ORA and GSEA likewise identified EMT as significantly dysregulated across PS-C treatments. Activation of EMT-related transcriptomic programs is associated with increased motility and reduced adhesion (Chen et al., 2017), consistent with the wound-healing phenotypes observed here. However, it should be noted that these EMT signatures are based on pathway-level enrichment and have not been confirmed by gain or loss of canonical EMT protein markers (e.g., E-cadherin, N-cadherin, vimentin). The association between transcriptomic EMT enrichment and functional outcomes should therefore be considered correlative rather than evidence of a complete endothelial-to-mesenchymal transition. Interestingly, the heatmap of EMT-related genes (Supplementary Figure S3C) showed the strongest alterations in PS-C 100 nm, which displayed more dysregulated genes compared to PS-C 30 and 50 nm.

Migration is a critical endothelial function involved in vascular repair and homeostasis, and its disruption could contribute to early atherosclerotic events (Li et al., 2020). The migration phenotypes observed here are consistent with cholesterol-trafficking alterations that can impact adhesion and cytoskeletal dynamics, supporting PS-NPL–driven remodelling of endothelial motility.

### 3.8 Angiogenesis of HUVECs exposed to PS-C-NPLs by tube formation assay

In the tube formation assay, PS-C-NPL exposure altered vascular network formation (Figures 5E,F). PS-C 100 nm increased covered area, branch points, and total tube length relative to the control, and PS-C 50 nm produced a similar but generally smaller enhancement. In contrast, PS-C 30 nm reduced the covered area with limited effects on other parameters. These outcomes indicate that PS-NPL exposure can remodel angiogenic behaviour, consistent with the broad transcriptional reprogramming observed by RNA-seq.

Angiogenesis is a multifactorial process that depends not only on migration but also on proliferation, differentiation, extracellular matrix remodeling, and coordinated signaling (Flournoy et al., 2022). Accordingly, changes in migration and tube formation are not necessarily coupled. In our study, angiogenesis phenotypes were supported by transcriptomic alterations in EMT- and angiogenesis-related gene sets (Supplementary Figures S3B,C), reinforcing the notion that PS-NPL exposure can rewire endothelial programs that control vascular remodelling. Further mechanistic work will be required to identify the proximal drivers, but the multilayer concordance presented here provides a functional interpretation of the molecular signatures.

### 3.9 Evaluation of IL-6 expression in HUVECs after PS-C-NPL exposure

**FIGURE 6:**
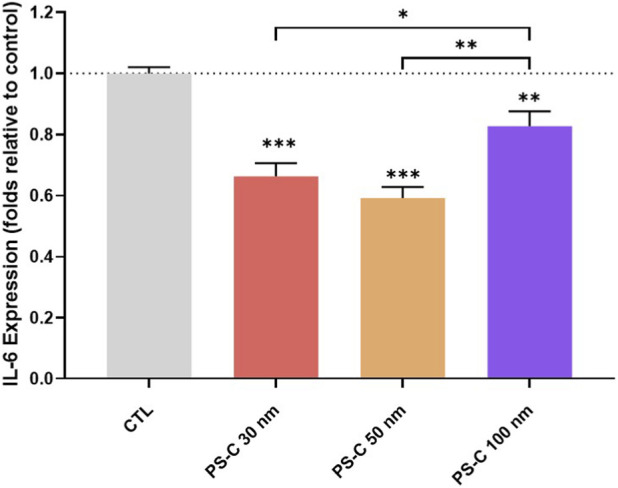
IL-6 expression in HUVECs exposed to PS-C-NPLs (100 μg/mL, 24 h). IL-6 levels were quantified after treatment and are shown as mean ± SEM. Statistical analysis was carried out using one-way ANOVA with Dunnett’s and Tukey’s post-test (parametric data). *p ≤ 0.05, **p ≤ 0.01, ***p ≤ 0.001.

IL-6 secretion was significantly reduced in HUVECs following exposure to PS-C-NPLs (Figure 6). The decrease was most pronounced under PS-C 50 nm (41% reduction), followed by PS-C 30 nm (34%) and PS-C 100 nm (17%). Although the magnitude differed, the consistent downregulation of IL-6 indicates an immunomodulatory effect of PS-NPL exposure in this endothelial model. This functional phenotype was consistent with the transcriptomic signatures: ORA revealed dysregulation of IL2–STAT5 signaling across treatments, and GSEA supported enrichment for inflammatory response programs in PS-C at 50/100 nm. Together, these results support that PS-NPL exposure reshapes inflammatory signaling at the pathway level, with downstream consequences for cytokine output.

Beyond immunomodulation, several complementary mechanisms may contribute to the observed reduction in IL-6. Given the extensive vesicular accumulation and endolysosomal alterations observed in our study, particularly for PS-C 50 and 100 nm, the cellular secretory machinery may be physically or functionally impaired, thereby retaining IL-6 intracellularly. Additionally, protein corona formation on nanoplastics could sequester secreted IL-6, reducing its detection by ELISA. This is supported by recent evidence showing that microplastics efficiently adsorb functional proteins within their biocorona, impairing their biological availability (Brouwer et al., 2025). The observed reduction may therefore reflect both transcriptional reprogramming and physical sequestration of the cytokine.

Additionally, significant IL-6 dysregulation across all treatments provides mechanistic support for the observed alterations in HUVEC migration and angiogenesis, in line with extensive evidence linking IL-6 to endothelial motility and angiogenic regulation. IL-6 enhances inflammatory cell migration by promoting cell adhesion and inducing chemokine production (Suzuki et al., 2010). Furthermore, it plays a pivotal role in angiogenesis by regulating the expression of angiopoietins and VEGF, key mediators of blood vessel formation (Kayakabe et al., 2012). Beyond migration and angiogenesis, IL-6 modulates fundamental cellular functions in HUVECs, including proliferation and inflammation, thereby contributing to endothelial dysfunction. Specifically, IL-6 has been associated with the regulation of chemokines, such as MCP-1 and IL-8, and with the expression of adhesion molecules, such as ICAM-1 (Kang and Kishimoto, 2021). These factors facilitate monocyte infiltration and migration, marking a critical early step in the initiation and pathogenesis of atherosclerosis (Ohta et al., 2020). IL-6 has been linked to atherosclerosis pathogenesis through its ability to increase endothelial lipase expression via the p38 MAPK and NF-κB signaling pathways (Yue et al., 2016). Consistent with this role, our transcriptomic analyses identified enrichment of the WikiPathways “Fluid Shear Stress and Atherosclerosis” and “Lipid and Atherosclerosis” pathways (data not shown). This molecular evidence parallels recent clinical findings from Marfella et al. (2024), who demonstrated that the presence of MNPLs in human carotid plaques was associated with a significantly increased risk of cardiovascular events, including myocardial infarction, stroke, and all-cause mortality.

## 4 Conclusion

This study provides a comprehensive evaluation of the endothelial effects of carboxylated polystyrene nanoplastics (PS-C-NPLs) in primary HUVECs, using an integrated design that couples transcriptomics with phenotypic endpoints. By combining RNA-seq bioinformatics (DEG, ORA, and GSEA) with particle-uptake imaging and functional assays, we move beyond descriptive screening to link pathway-level alterations with measurable changes in endothelial homeostasis.

Across treatments, HUVECs rapidly internalized PS-C-NPLs and exhibited extensive intracellular accumulation. Transcriptomic analyses revealed a dominant core program characterized by dysregulation of cholesterol homeostasis, inflammatory and stress signaling, endothelial plasticity (EMT), and genome integrity pathways. Guided by these molecular signatures, functional assays confirmed intracellular cholesterol accumulation, DNA damage, changes in migratory capacity and angiogenic behaviour, and reduced IL-6 secretion. The concordance between RNA-seq-derived pathways and functional readouts supports a mechanistic framework in which PS-NPL exposure rewires endothelial metabolic and stress-response networks with downstream consequences for vascular function.

These findings highlight the potential for PS-NPL exposure to contribute to endothelial dysfunction, a key early event in vascular pathology. More broadly, the study demonstrates the value of bioinformatics as a mechanistic layer: transcriptomic programs were not merely catalogued but used to guide, interpret, and contextualize functional outcomes. While nanoscale differences modulated specific response features, the shared molecular core response provides a coherent basis for understanding PS-NPL-driven endothelial disruption and for prioritizing mechanistically anchored endpoints in future hazard assessment.

To extend this work, future studies should prioritize dose–response characterization at lower, environmentally realistic concentrations to establish quantitative thresholds for the molecular and functional endpoints identified here. Additional priorities include exploring other polymers such as polyethylene terephthalate or polylactic acid and employing more advanced experimental models, including endothelial barriers or tissue-engineered vessels. Such efforts may help refine our understanding of nanoplastic toxicity and its cardiovascular implications.

## Data Availability

The original contributions presented in the study are publicly available. This data can be found at Gene Expression Omnibus with the accession number GSE324731.
