# Supplementary material for: Transcriptomic and functional profiling of endothelial dysfunction induced by polystyrene nanoplastics
Source: Front Toxicol. 2026 Apr 17;8:1812922. doi: 10.3389/ftox.2026.1812922 (PMC13132504; doi:10.3389/ftox.2026.1812922)
Supplement: Supplementary file 1 [file DataSheet1.docx]

Supplementary Material

# Transcriptomic and functional profiling of endothelial dysfunction induced by polystyrene nanoplastics

Joan Martín-Pérez, Aliro Villacorta, Javier Gutierrez-García, Raquel Egea, Michelle Morataya-Reyes, Mireia Cassú-Casadevall, Irene Barguilla, Ricard Marcos, Alba Hernández*, Alba García-Rodríguez*

## Supplementary Figures


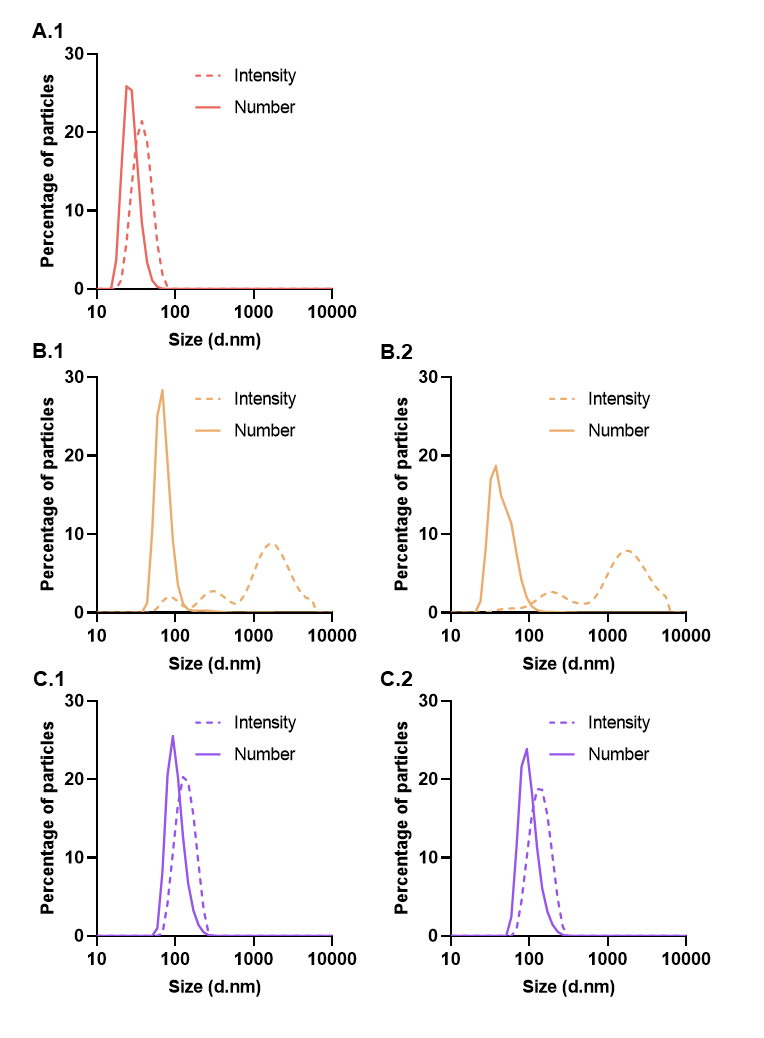


**Supplementary Figure S1.** Size distribution of PS-C-NPLs in EGM-2 medium measured by DLS. The graphs show the size distribution as a percentage of particles by number (solid line) and intensity (dotted line) for the different PS-C-NPL sizes. (A.1) PS-C 30 FL, (B.1) PS-C 50 FL, (B.2) PS-C 50 NL, (C.1) PS-C 100 FL, and (C.2) PS-C 100 NL.

**Supplementary Figure S2.** Cell viability effects of different sizes and concentrations of PS-C-NPLs in HUVECs after 24 hours of treatment. Viability is represented as the percentage of viability relative to the negative control (untreated cells).

**
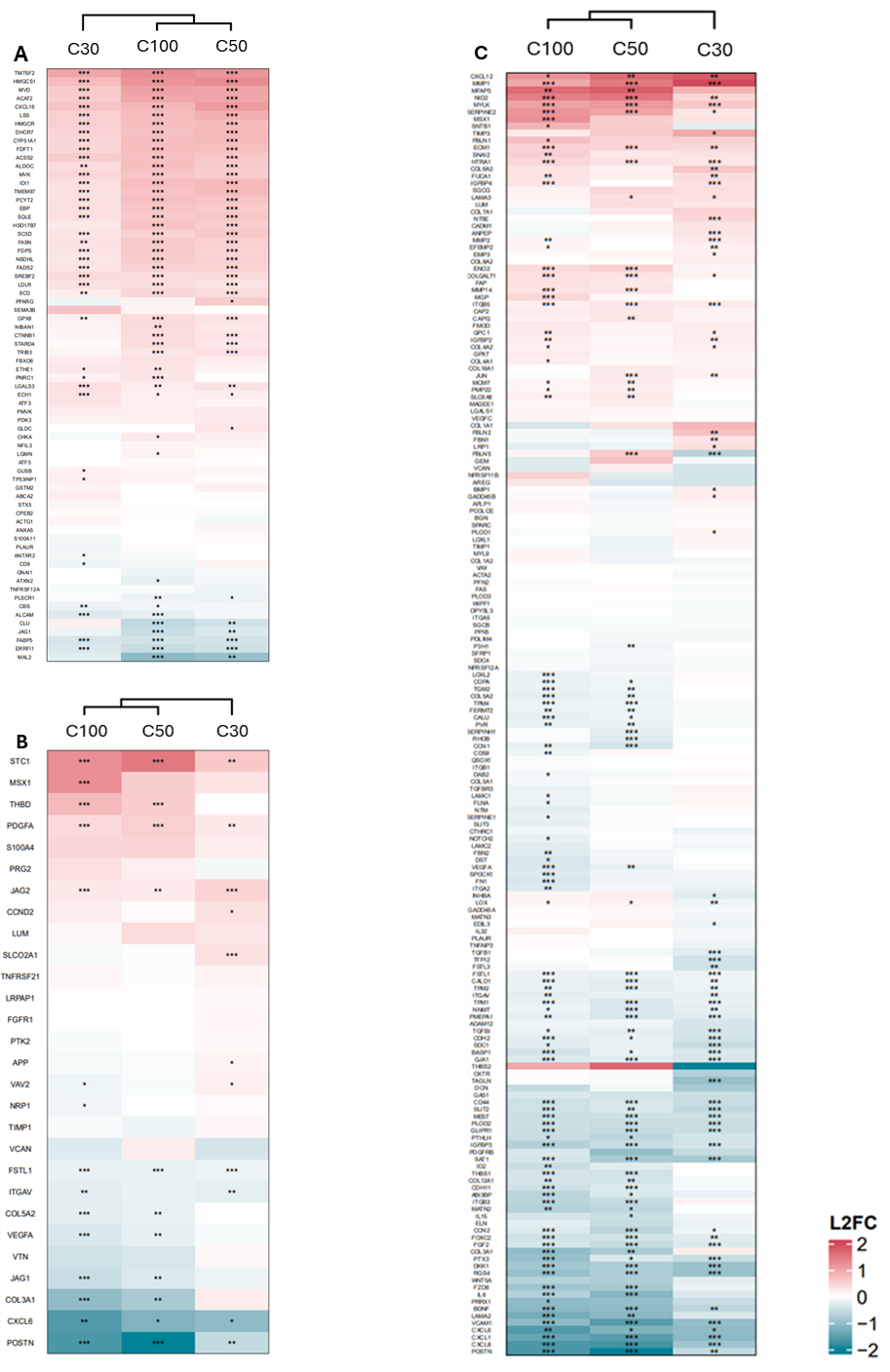
**

**Supplementary Figure S3.** Heatmaps for different MSigDB hallmark gene sets, including: Cholesterol Homeostasis (A), Angiogenesis (B), and Endothelial-Mesenchymal Transition (C), with dendrograms to cluster the treatments according to their gene expression patterns. Gene expression values are represented as log2 fold-change values (L2FC). Statistical significance is denoted as **p* ≤ 0.05, ***p* ≤ 0.01, and ****p* ≤ 0.001.
